# Prognostic utility of preoperative inflammatory markers in patients with intrahepatic cholangiocarcinoma after hepatic resection: A systematic review and meta‐analysis

**DOI:** 10.1002/cam4.4935

**Published:** 2022-06-12

**Authors:** Hongxia Cui, Yarong Li, Su Li, Guangxuan Liu

**Affiliations:** ^1^ Department of Pharmacy Cancer Hospital of China Medical University, Liaoning Cancer Hospital and Institute Shenyang China; ^2^ School of Life Science and Biopharmaceutics Shenyang Pharmaceutical University Shenyang China

**Keywords:** inflammatory biomarkers, intrahepatic cholangiocarcinoma, meta‐analysis, prognosis

## Abstract

**Background:**

The prognostic value of preoperative systemic inflammatory markers, including the neutrophil‐to‐lymphocyte ratio (NLR), platelet‐to‐lymphocyte ratio (PLR), and lymphocyte‐to‐monocyte ratio (LMR), remains controversial in patients with intrahepatic cholangiocarcinoma (ICC). Therefore, this meta‐analysis aimed to investigate the prognostic value of preoperative NLR, PLR, and LMR in patients with ICC who underwent hepatic resection.

**Methods:**

We conducted a comprehensive search of four electronic databases. Two researchers assessed the quality of the available data using the Newcastle–Ottawa Scale. We selected overall survival (OS) as the primary outcome and recurrence‐free survival (RFS) and disease‐free survival (DFS) as secondary outcomes. Hazard ratios (HRs) and 95% confidence intervals (CIs) were merged to evaluate the associations between inflammatory markers and ICC patient prognosis.

**Results:**

Fifteen studies (18 cohorts) with 4123 cases were included in this meta‐analysis. The results revealed that a high preoperative NLR was associated with short OS and RFS (HR = 1.04, 95% CI: 1.01–1.07, and HR = 1.29, 95% CI: 1.04–1.60, respectively) in patients with ICC. However, the association between PLR or LMR and ICC prognosis was not statistically significant. In addition, the publication bias and sensitivity analyses demonstrated that the results were reliable and stable.

**Conclusion:**

Our meta‐analysis revealed that preoperative NLR may be a useful prognostic predictor for patients with ICC.

## Graphical abstract

This meta‐analysis aimed to investigate the prognostic value of preoperative inflammatory markers in patient with intrahepatic cholangiocarcinoma (ICC) who underwent hepatic resection. Our study revealed that high preoperative neutrophil‐lymphocyte ratio (NLR) is associated with worse prognosis in patients with ICC. The results from our meta‐analysis suggest that NLR may be used as a potential prognostic predictor for patients with ICC.

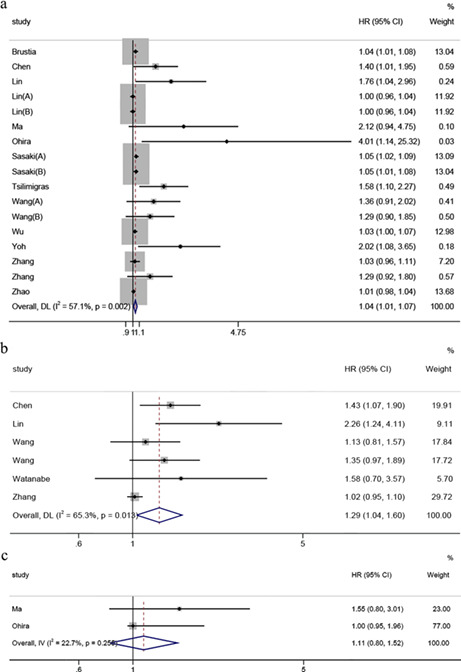

## 1 BACKGROUND

Intrahepatic cholangiocarcinoma (ICC) is one of the most common types of primary liver cancer, accounting for 10%–15% of all primary liver cancer cases.^1^ In recent years, its incidence has increased significantly worldwide.^2^, ^3^ The most effective radical treatment is hepatectomy, but most patients with ICC are not fit to undergo surgery upon diagnosis with ICC.^4^ For patients with operable ICC, the median survival after curative‐intent resection is 24–36 months, which is still unsatisfactory.^5^, ^6^, ^7^ Although most patients with ICC receive the same treatment, their clinical outcomes may differ due to tumor heterogeneity and various systemic factors. Therefore, it is necessary to identify factors that can predict the prognosis and aid clinicians in selecting the most suitable therapeutic strategies for patients with ICC.

Inflammation is a hallmark of cancer.^8^ Increasing evidence indicated that cancer‐associated inflammations are involved in numerous cancer‐related processes, including cancer initiation, progression, and metastasis.^9^ Neutrophil‐lymphocyte ratio (NLR) is defined as the ratio of absolute neutrophil count to absolute lymphocyte count, platelet‐to‐lymphocyte ratio (PLR) is defined as the ratio of absolute thrombocyte count to absolute lymphocyte count, and lymphocyte‐to‐monocyte ratio (LMR) is defined as the ratio of absolute lymphocyte count to absolute monocyte count. These blood biomarkers reflect the inflammatory status^10^ and have been proved to be valuable in predicting the prognosis of many cancer types, including colorectal cancer,^11^ breast cancer,^12^ urinary neoplasm,^13^ and esophagogastric junction cancer.^14^

The associations between NLR, LMR, PLR, and clinical outcomes in patients with ICC have been explored in various studies, but there is no consensus in results.^15^, ^16^, ^17^, ^18^, ^19^, ^20^, ^21^, ^22^, ^23^, ^24^, ^25^, ^26^, ^27^, ^28^, ^29^ Two previous meta‐analyses evaluated the role of inflammatory factors in predicting the prognosis of patients with cholangiocarcinoma.^30^, ^31^ However, they focused on the whole cholangiocarcinoma cohort, not specifically on patients with ICC. In addition, they did not exclude the impact of preoperative treatment on the clinical outcomes of patients. Therefore, in this study, we assessed the predictive values of preoperative NLR, PLR, and LMR in patients with ICC who did not receive preoperative therapy by performing a meta‐analysis.

## 2 METHODS

The protocol for this meta‐analysis was registered in PROSPERO (CRD42021250132). We followed the Preferred Reporting Items for Systematic Reviews and Meta‐analysis (PRISMA) guidelines for this meta‐analysis.^32^

### 2.1 Search strategy

We conducted a comprehensive search of PubMed, Cochrane Library, EMBASE, and Web of Science using a combination of relevant keywords and medical subject heading terms. The main keywords were as follows: biliary tract neoplasms, cholangiocarcinoma, neutrophil‐to‐lymphocyte ratio, PLR, LMR, NLR, PLR, LMR, prognosis, overall survival, recurrence‐free survival, disease‐free survival, OS, RFS, and DFS. File S1 describes the detailed methods used to search PubMed. All databases were searched from their inception to May 2021. We also manually searched the references of each relevant article to identify more suitable articles.

### 2.2 Selection criteria

The inclusion criteria were as follows: (1) P: patients in the original studies were diagnosed with ICC; (2) I: ICC group with high preoperative NLR, PLR, and LMR; (3) C: ICC group with low preoperative NLR, PLR, and LMR; (4) O: studies that reported the association between NLR, PLR, LMR, and overall survival (OS), recurrence‐free survival (RFS), and disease‐free survival (DFS) of patients with ICC; and (5) S: prospective or retrospective cohort studies. Exclusion criteria were as follows: (1) patients who underwent preoperative therapies; (2) studies that provided insufficient information for extracting hazard ratios (HRs) and 95% confidence intervals (CIs); (3) reviews, conference abstracts, comments, case reports, and letters; (4) duplicated studies or publications; and (5) nonhuman studies

### 2.3 Data extraction

Two researchers selected eligible articles and extracted the following information: author, country, publication year, type of research, basic characteristics of patients, tumor type, treatment strategy, tumor stage, cutoff values of inflammatory markers, study endpoints, HRs, and 95% CIs for OS, RFS, and DFS. When HRs obtained from univariate and multivariate analyses were both reported, we selected the HRs obtained from multivariate analysis as multivariate analysis can exclude correlated confounding factors and is more accurate. If some original articles did not report HRs directly, Engauge Digitizer software (version 4.1) was used to extract the HRs from survival curves. The two researchers reached consensus after discussing all differences.

### 2.4 Quality assessment

The quality of each included study was evaluated using the Newcastle–Ottawa Scale (NOS). The NOS criteria included three items: (1) selection, (2) comparability, and (3) outcome (cohort study).^33^ The highest NOS score was 9, and studies with a score of ≥6 were considered to be high‐quality studies.

### 2.5 Statistical analysis

We selected OS as the primary outcome and RFS and DFS as the secondary outcomes. We combined the HRs and 95% CIs of each included study to evaluate the influence of preoperative NLR, PLR, and LMR on the prognosis of patients with ICC. We tested heterogeneity among the enrolled studies using Cochrane's Q (Chi squared) and I^2^ statistic. If *I* ^2^ < 50% or *p* > 0.10, indicating that significant heterogeneity did not exist, then HRs and 95% CIs were merged with a fixed effects model; otherwise, a random‐effects model was selected. Subgroup analyses based on statistical methods (multivariate or univariate) and sample sizes (<200 or ≥200) were performed to identify the cause of heterogeneity. In addition, Egger's test and Begg's test were performed for the evaluation of publication bias, and the trim‐and‐fill method was used to adjust for publication bias. A sensitivity analysis was conducted to determine the robustness of the synthesized results. Statistical analyses were performed using Stata 14.0 (Stata Corp.).

## 3 RESULTS

### 3.1 Selection of studies

**FIGURE 1:**
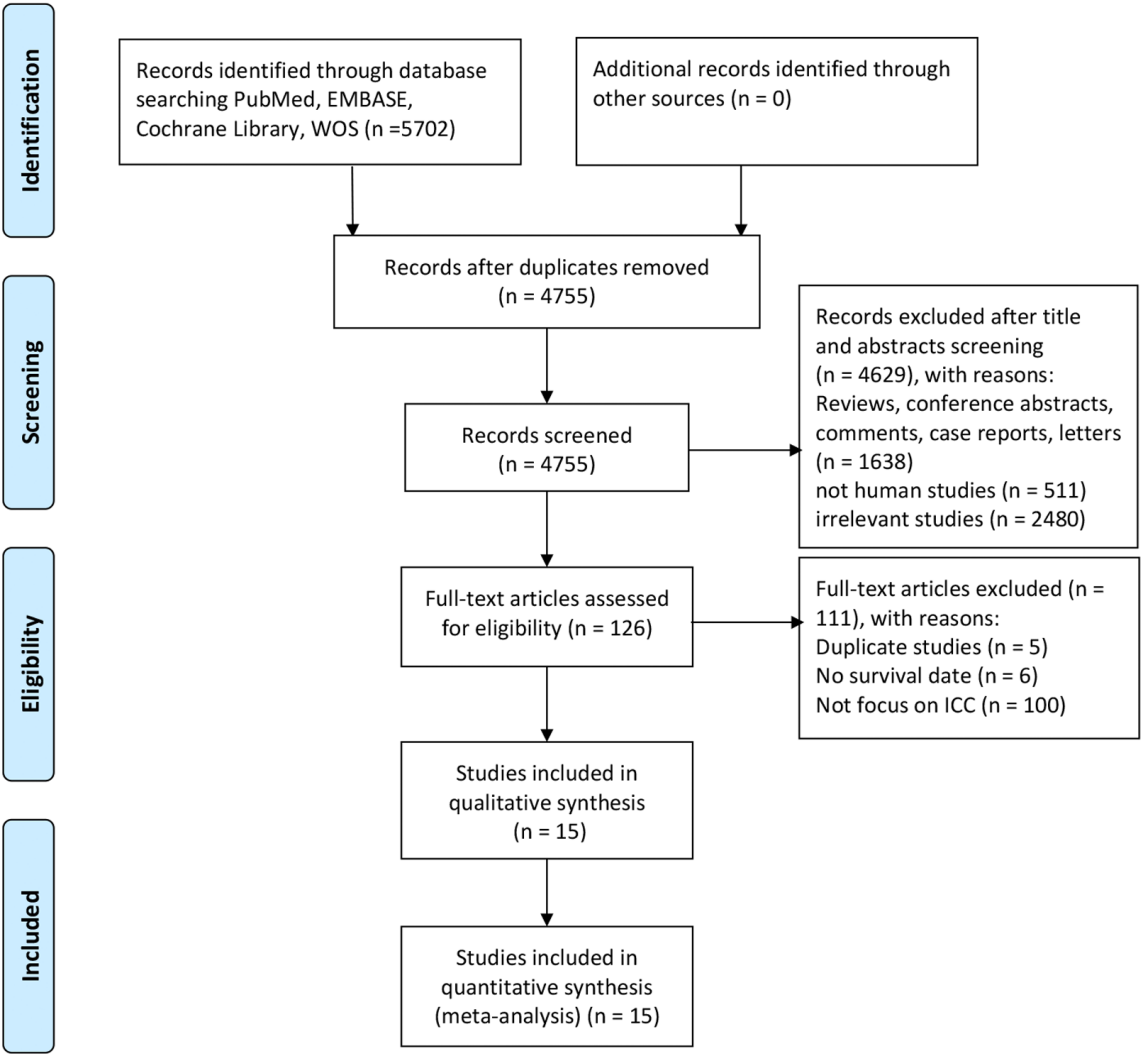
Procedure of literature screening

Based on the selection criteria, 5702 articles were retrieved. First, 947 duplicates were removed. We then browsed the titles and abstracts of these studies and excluded 4629 studies if they included reviews, conference abstracts, comments, case reports, letters, nonhuman studies, and irrelevant studies. From 126 studies selected for full‐text examination, 111 were excluded owing to the following selection criteria: duplicate studies (*n* = 5), no survival date (*n* = 6), and no focus on ICC (*n* = 100). Finally, 15 studies (18 cohorts) [15‐29]with 4123 patients were included in our meta‐analysis. Figure 1 is the process of literature screening.

### 3.2 Characteristics of included studies

The main features of the selected 15 studies are listed in Table 1. Notably, among these 15 studies, three contained simultaneous training and validation cohorts. Therefore, we divided each of the three studies into two separate cohorts and finally conducted a comprehensive analysis of 18 cohorts. The included cohorts were published between 2015 and 2021, with 11 cohorts from China, three from international multi‐centers, two from Japan, one from America, and one from Singapore. The sample size of each study ranged from 44 to 688. All cohorts determined the prognostic value of NLR, seven cohorts studied the relationship between PLR and prognosis, and 12 cohorts explored the predictive value of LMR. All studies were retrospective in nature. The NOS scores of all cohorts ranged from 7 to 8, suggesting good quality.

**TABLE 1:** Major characteristics of eligible studies

Author	Year	Country	Stage	No. of pts	Age (y)	Tumor type	Markers	Cutoff value			Outcome	Study design	Analysis method	Treatment	NOS score
								NLR	PLR	LMR					
Brustia^15^	2019	International multi‐centered	I–IV	355	68 (60–75)^a^	ICC	NLR	NR	NR	NR	OS	Retrospective	MV	Surgery + adjuvant therapy	8
Chen^16^	2015	China	I–IVa	322	57.8 ± 11.2^a^	ICC	NLR, LMR	NR	123	NR	OS/RFS	Retrospective	MV	NR	8
Lin^29^	2015	China	I–IV	102	NR	ICC	NLR	3	NR	NR	OS/RFS	Retrospective	MV	Surgery	8
Lin(A)^17^ TC	2019	China	I‐IV	123	60 (31–85)^a^	ICC	NLR, PLR, LMR	2.94	130.6	3.62	OS/DFS	Retrospective	MV/UV	NR	8
Lin(B)^17^ VC	2019	China	I‐IV	95	61 (37–79)^a^	ICC	NLR, PLR, LMR	2.94	130.6	3.62	OS/DFS	Retrospective	UV	NR	8
Ma^18^	2021	China	I‐IV	102	49 (28–77)^a^	ICC	NLR, PLR, LMR	3	90	2.7	OS/DFS	Retrospective	MV/UV	NR	8
Ohira^19^	2019	Singapore	I‐IV	52	NR	ICC	NLR, PLR, LMR	1.93	98	4.36	OS/DFS	Retrospective	MV/UV	Surgery + adjuvant therapy	8
Sasaki(A)^20^ TC	2017	International multi‐centered	I–IV	269	58 (51–66)^a^	ICC	NLR	NR	NR	NR	OS	Retrospective	MV	NR	8
Sasaki(B)^20^ VC	2017	International multi‐centered	I–IV	269	57 (49–64)^a^	ICC	NLR	NR	NR	NR	OS	Retrospective	MV	NR	8
Tsilimigras^21^	2020	America	I‐IV	688	57 (49–65)^a^	ICC	NLR, PLR	5	190	NR	OS	Retrospective	MV	Surgery + adjuvant therapy	8
Wang^22^ TC	2020	China	I‐III	264	57.26 ± 10.71^b^	ICC	NLR, LMR	2.62	103	NR	OS/RFS	Retrospective	MV	NR	8
Wang^22^ VC	2020	China	I‐III	263	57.26 ± 10.72^b^	ICC	NLR, LMR	2.62	103	NR	OS/RFS	Retrospective	MV	NR	8
Watanabe^23^	2019	Japan	I‐IV	44	46–88^b^	ICC	NLR	3	NR	NR	RFS	Retrospective	UV	NR	7
Wu^24^	2018	China	I‐IV	123	56.8 ± 10.67^b^	ICC	NLR, LMR	2.05	NR	3.42	OS	Retrospective	MV	NR	8
Yoh^25^	2017	Japan	I‐IV	134	65 (26–84)^a^	ICC	NLR, LMR	5	120	NR	OS	Retrospective	MV	Surgery + adjuvant therapy	8
Zhang^27^	2020	China	I–III	128	56.19 ± 9.63^b^	ICC	NLR, PLR, LMR	3.3	156.8	3.2	OS/RFS	Retrospective	MV/UV	Surgery	8
Zhang^26^	2018	China	I–IVa	322	57.9 (27–81)^b^	ICC	NLR, LMR	NR	NR	4.45	OS	Retrospective	MV	NR	8
Zhao^28^	2021	China	I–IVa	468	58 (51–65)^a^	ICC	NLR, PLR, LMR	NR	143.5	NR	OS	Retrospective	MV/UV	Surgery + adjuvant therapy	8

Abbreviations: MV, multivariate analysis; NOS, Newcastle–Ottawa Scale; NR, not reported; TC, training cohort; UV, univariate analysis; VC, validation cohort.

^a^ Median.

^b^ Mean.

### 3.3 Prognostic value of NLR

**FIGURE 2:**
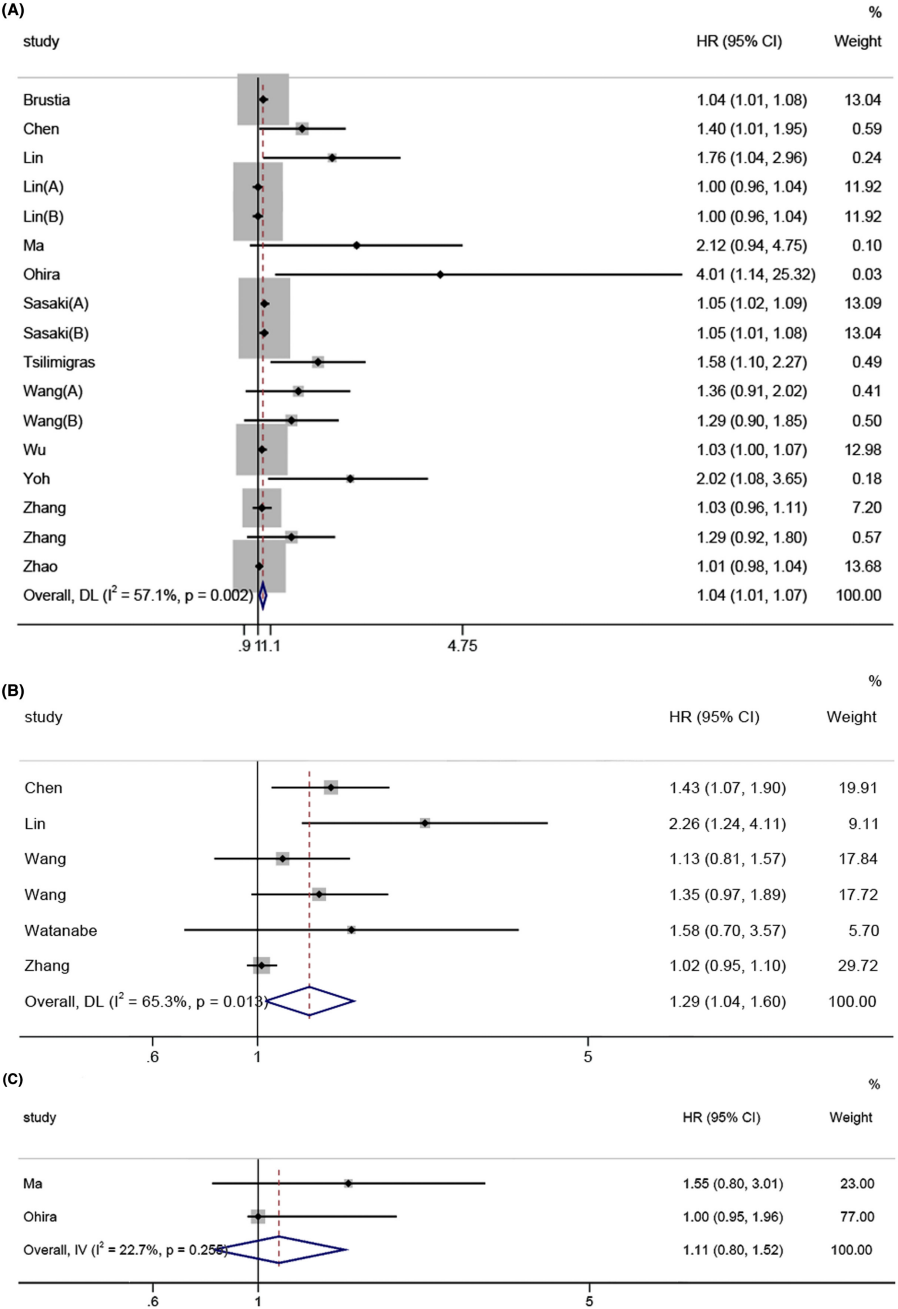
Forest plot for association between NLR and survival in ICC. (a) OS; (b) RFS; (c) DFS

Seventeen cohorts reported the predictive value of NLR for OS. Due to the presence of moderate heterogeneity between studies (*I* ^2^ = 57.1%, *p* = 0.001), HRs were pooled using a random‐effects model. The pooled results revealed that a high preoperative NLR was significantly associated with poorer OS in patients with ICC (HR = 1.04, 95% CI: 1.01–1.07, Figure 2A). To further identify the potential factors for heterogeneity, we performed subgroup analyses stratified by statistical methods and sample size. Subgroup analyses showed that statistical methods could slightly reduce the heterogeneity. NLR was still significantly associated with OS in studies analyzed using the multivariate method (HR = 1.07, 95% CI: 1.03–1.11) and in studies with a sample size ≥200 (HR = 1.05, 95% CI: 1.01–1.08). In contrast, NLR did not have a significant prognostic effect on OS in studies analyzed by univariate method (HR = 1.00, 95% CI: 0.98–1.02), in studies with sample size <200 (HR = 1.03, 95% CI: 0.98–1.07). The details are presented in Table 2.

**TABLE 2:** Subgroup analysis of NLR for OS in ICC

Factor	No. of studies	No. of pts	HR and 95%CI	Heterogeneity	
				*I* ^2^ (%)	*p*‐value
OS	17	4079	1.04(1.01–1.07)	57.1	0.002
Statistical method					
Multivariate	13	3265	1.07 (1.03–1.11)	55.7	0.008
Univariate	4	814	1.00 (0.98–1.02)	0	0.884
Sample size					
<200	8	859	1.03 (0.98–1.07)	60.3	0.014
≥200	9	3220	1.05 (1.01–1.08)	56.0	0.020

Six cohorts reported data on the association between the NLR and RFS.^16^, ^22^, ^23^, ^26^, ^29^ With observable heterogeneity in the six studies (*I* ^2^ = 65.3%, *p* = 0.013), we adopted a random‐effects model. The emerged HR was 1.29 (95% Cl: 1.04–1.60; Figure 2B), indicating that a high preoperative NLR significantly predicted shorter RFS in patients with ICC.

Only two cohorts studied the prognostic effect of NLR on DFS. We did not observe significant heterogeneity between the studies (*I* ^2^ = 22.7%, *P* = 0.255); therefore, a fixed‐effects model was employed. The combined results implied that NLR did not have a predictive value for DFS (HR = 1.11, 95% CI: 0.80–1.52; Figure 2c).

### 3.4 Prognostic value of PLR

Eleven studies assessed the relationship between the PLR and OS. Significant heterogeneity was detected (*I* ^2^ = 66.6%, *p* = 0.001); therefore, we pooled the HRs using a random‐effects model. The pooled results revealed that PLR was not related to OS (HR = 1.00, 95% CI: 0.99–1.01, Figure S1). To further identify the potential factors for heterogeneity, we performed subgroup analyses stratified by statistical methods and sample size. Subgroup analyses showed that the sample size could slightly reduce the heterogeneity. PLR was not related to OS, regardless of statistical methods or sample size. The results are summarized in Table 3.

**TABLE 3:** Subgroup analysis of PLR for OS in ICC

Subgroup	Number of studies	No. of pts	HR and 95% CI	Heterogeneity	
				*I* ^2^ (%)	*p*‐value
OS	11	2833	1.00 (0.99–1.01)	66.6	0.001
Statistical method					
Multivariate	7	372	1.00 (1.00–1.01)	79.8	0.000
Univariate	4	2461	1.00 (0.98–1.02)	0	0.987
Sample size					
<200	6	506	1.00 (0.99–1.01)	0	0.265
≥200	5	2327	1.00 (1.00–1.01)	30.4	0.000

Four studies analyzed the predictive effect of PLR on RFS and only two studies supplied data on the association between PLR and DFS. We noted significant heterogeneity between studies for RFS (*I* ^2^ = 66.1%, *p* = 0.032) and DFS (*I* ^2^ = 72.8%, *p* = 0.055). Analysis with the random‐effects model suggested that PLR had no predictive effect on RFS (HR = 1.06, 95% CI: 0.87–1.29; Figure S2) or DFS (HR = 1.25, 95% CI: 0.70–2.24; Figure S3).

### 3.5 Prognostic value of LMR

Eight studies provided the association between the LMR and OS. Given the existence of heterogeneity (*I* ^2^ = 65.4%, *p* = 0.005), a random‐effects model was employed. No significant difference was observed in the association between LMR and OS (HR = 0.99, 95% CI: 0.93–1.05; Figure S4). In the subgroup analyses of statistical methods and sample size, we observed that the above two factors did not reduce heterogeneity. In addition, LMR was not related to OS, regardless of statistical methods or sample size. The results are summarized in Table 4.

**TABLE 4:** Subgroup analysis of LMR for OS in ICC

Subgroup	Number of studies	No. of pts	HR and 95%CI	Heterogeneity	
				*I* ^2^ (%)	*p*‐value
OS	8	1413	0.99 (0.93–1.05)	65.4	0.005
Statistical method					
Multivariate	5	722	0.82 (050–1.35)	76.4	0.002
Univariate	3	691	1.00 (1.00–1.01)	0	0.951
Sample size					
<200	6	623	0.96 (0.81–1.14)	74.9	0.001
≥200	2	790	1.00 (0.99–1.01)	0	0.608

Only one study reported an association between LMR and RFS; therefore, we did not merge these data. Next, we included two studies that evaluated the association between LMR and DFS. No heterogeneity was found (*I* ^2^ = 0%, *p* = 0.534); therefore, we adopted the fixed‐effects model for analysis. The synthesized HR was 1.00 (95% Cl: 0.95–1.05; Figure S5), indicating that LMR had no predictive value for DFS in patients with ICC.

### 3.6 Publication bias

**FIGURE 3:**
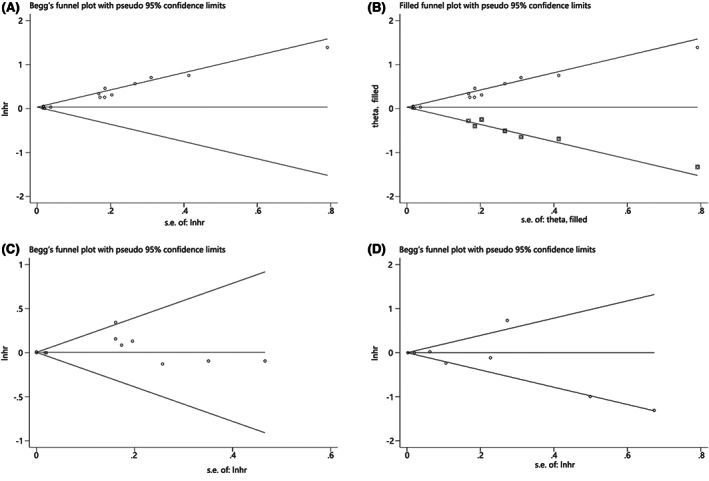
Funnel plot for publication bias. (a) Begg's funnel plot for OS of NLR; (b) Trim‐and‐fill funnel plot for OS of NLR; (c) Begg's funnel plot for OS of PLR; (d) Begg's funnel plot for OS of LMR.

Regarding the publication bias of NLR for OS, the *p*‐value of Begg's test was 0.007, and the *p*‐value of Egger's test was 0.000, suggesting the presence of publication bias (Begg's test, Figure 3A). Subsequently, we evaluated the stability of the combined HR value using the trim‐and‐fill method. The recombined result confirmed that NLR was still a useful predictive marker for OS (HR = 1.03, 95% CI: 1.02–1.04; Figure 3B), which did not differ from the initial result. Additionally, we found no significant publication bias of PLR for OS (*p* = 0.917 for Begg's test, Figure 3c; and *p* = 0.218 for Egger's test) or LMR for OS (*p* = 0.536 for Begg's test, Figure 3d; and *p* = 0.447 for Egger's test), indicating the robustness of our meta‐analysis.

### 3.7 Sensitivity analysis

**FIGURE 4:**
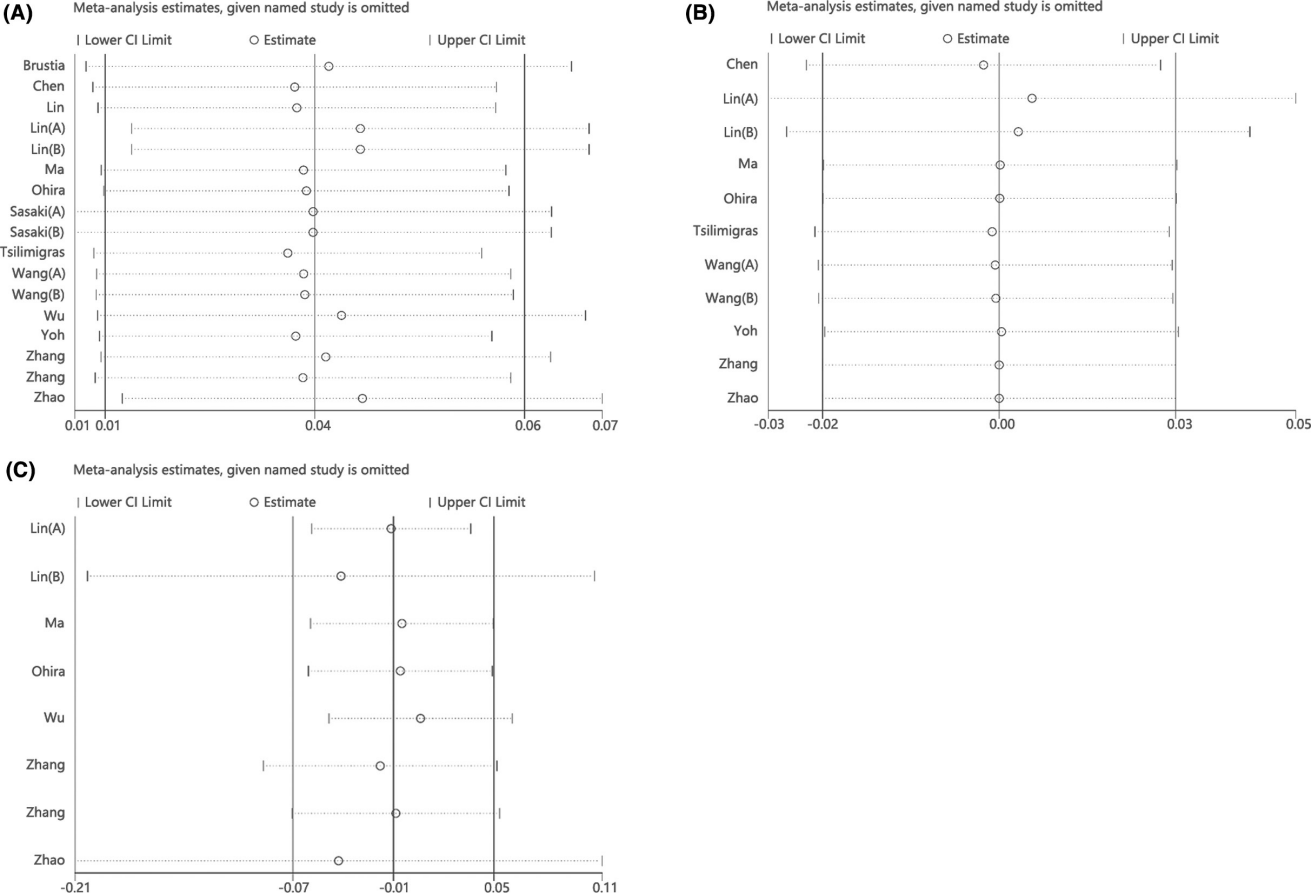
Sensitivity analysis. (a) NLR and OS; (b) of PLR and OS; (c) LMR and OS

We sequentially removed each study to assess its impact on the combined result. Sensitivity analysis revealed that the associations of OS with NLR, PLR, and LMR were not significantly changed in any independent study (Figure 4A–C), which supports the results of our meta‐analysis.

## 4 DISCUSSION

Cancer‐related inflammation is associated with tumor growth, progression, and metastasis.^8^, ^9^, ^34^ NLR, PLR, and LMR are of great significance in determining the patient prognosis in various tumors.^35^, ^36^, ^37^ Although there are many studies based on the association between these indices and ICC patient prognosis, the results are not uniform. In our meta‐analysis, we summarized the results of 15 studies (18 cohorts) to systematically assess the prognostic value of preoperative NLR in patients with ICC. Our results showed that a high NLR was associated with short OS and RFS, but PLR and LMR were not associated with OS, RFS, and DFS. Furthermore, subgroup analyses suggested that patients with a high NLR had a short OS than those with a low NLR in studies analyzed using the multivariate method and in studies with a sample size ≥200. Moreover, the publication bias and sensitivity analyses demonstrated the reliability and stability of our study. Collectively, preoperative NLR can be used as an important prognostic predictor for patients with ICC. As NLR can be easily determined via routine blood tests, this parameter may have great clinical application potential to guide decision‐making in clinical settings.

Although the NLR can serve as a prognostic biomarker of ICC, the specific mechanism remains unclear. Considering that NLR is the ratio of neutrophils to lymphocytes, we speculate that the potential mechanism may be related to the functions of neutrophils and lymphocytes. In general, neutrophils are involved in every step of carcinogenesis, including tumor initiation, growth, and metastasis. Specifically, during tumor initiation, neutrophils can produce reactive oxygen species (ROS), matrix metalloprotein (MMP9), and reactive nitrogen species (RNS), which further promote tumor initiation.^38^ Neutrophils can promote tumor growth by inducing angiogenesis or by compromising immunity via amino acid consumption or release of specific cytokines.^39^ At the site of metastasis, neutrophils can restrict the antitumor function of CD8^+^ T cells by generating inducible nitric oxide synthase (iNOS).^40^ Moreover, regulatory B cells instruct neutrophils to restrict the response of natural killer cells (NK cells) and T cells to metastatic lesions.^41^, ^42^ In contrast, lymphocytes, which mainly generate T lymphocytes, NK cells, and B cells, can induce tumor cell death and inhibit tumor proliferation and migration.^43^ More specifically, the antitumor response of lymphocytes is mainly mediated by the interaction between CD8^+^ and CD4^+^ T cells.^44^ CD8^+^ T cells, which directly kill tumor cells upon contact by expressing death ligands, can secrete cytotoxic mediators (perforin) and cytokines (interferon‐γ and tumor necrosis factor‐α).^45^ CD4^+^ T cells can release interleukin (IL)‐2, IL‐4, and IL‐5, which can activate B cells, cytotoxic T cells, and macrophages.^46^ In addition, accumulating evidence has also demonstrated that the greater the number of tumor‐infiltrating lymphocytes, the better is the prognosis of patients.^47^, ^48^ According to the above mechanism, NLR is obtained by dividing the number of neutrophils with that of lymphocytes, and an increase in NLR can reflect an increase in the neutrophil‐dependent inflammatory response or a decrease in the lymphocyte‐mediated antitumor immune response, resulting in the poor prognosis of patients.

Subgroup analysis of NLR and OS showed that the statistical methods only slightly reduced the heterogeneity, and several sources of heterogeneity were speculated. First, heterogeneity may be caused by differences in the study regions. The included cohorts were from different regions (14 from Asia, one from America, three from multi‐centers involving America, Europe, Asia, and Oceania). The multicenter studies included different regions, such as America and Asia, and did not provide information on the specific number of people from each region. In addition, only one American study was available; therefore, a subgroup analysis based on regions could not be carried out.

Second, different types of postoperative treatment may be a potential source of heterogeneity. Although all the original studies excluded patients who had received preoperative treatment, the postoperative treatment strategies may have been different, resulting in heterogeneity in the meta‐analysis. Few studies have provided a postoperative treatment scheme; only five studies reported that some patients had received postoperative adjuvant chemotherapy, so subgroup analysis based on postoperative treatment methods cannot be completed. We suggest that detailed postoperative treatment methods should be provided in future studies.

Third, the macroscopic types of ICC may cause heterogeneity. Uchiyama et al.^49^ reported that the macroscopic types of ICC were associated with prognosis; however, the original 15 articles (18 cohorts) rarely considered macroscopic types of ICC. To decrease the heterogeneity from different macroscopic types, we suggest that future studies can limit the subjects to patients with a certain macroscopic type or provide sufficient subgroup data of each macroscopic type.

The strength of our meta‐analysis was that we excluded the interference of confounding factors. Patients may be exposed to corticosteroids or antibiotics after surgery, which may affect the levels of neutrophils and lymphocytes.^50^ In addition, stress during operation may also affect systemic inflammation.^51^ All studies enrolled in this meta‐analysis focused on preoperative blood samples, which excluded the effect of surgery on systemic inflammatory indicators. Moreover, all patients in the original studies did not receive any preoperative treatment, which eliminated the impact of drugs on the results of routine blood examinations. Finally, to our knowledge, previous meta‐analyses reported the prognostic role of one or two inflammatory factors in cholangiocarcinoma, while our meta‐analysis systematically summarized the potential clinical value of three inflammatory markers in ICC.

Several limitations for this meta‐analysis should be considered. First, our meta‐analysis was preliminary as only a small number of articles were included in this study; therefore, more high‐quality studies are needed to further evaluate the prognostic role of preoperative inflammatory markers in patients with ICC. Second, although the types of studies were not a part of the selection criteria, all the original studies included here were retrospective in nature, which may have caused a selection bias. Third, because the HR values were not reported in some original studies, we indirectly extracted the relevant data from the survival curves, which may have resulted in some bias. Fourth, most studies included in the present meta‐analysis were conducted in Asia. Owing to the differences in the genetic background, environment, and lifestyles of patients from various regions, the limited regions may have affected the reliability of our findings. Finally, the selected original studies did not have uniform cutoff values for NLR, PLR, or LMR; therefore, more large‐scale prospective studies are necessary to establish optimal cutoff values for these indicators.

## 5 CONCLUSION

In conclusion, our study revealed that high preoperative NLR is associated with worse prognosis in patients with ICC. Our results suggest that NLR may be used as a potential prognostic predictor for patients with ICC. Moreover, clinicians can combine the information on inflammatory markers with that on the TNM stage and histological subtype to predict the prognosis of patients with ICC.

## AUTHOR CONTRIBUTIONS

HC and YL designed the study and wrote the main manuscript text. HC, YL, and SL selected publications and analyzed the data. HC and YL evaluated study quality. SL and GL revised manuscript. All authors approved the final manuscript.

## CONFLICT OF INTEREST

The authors have declared no conflict of interest.

## Supporting information


Figure S1

Figure S2

Figure S3

Figure S4

Figure S5

File S1

## Data Availability

The data that support the findings of this study are available from the corresponding author upon reasonable request.
