# Supplementary figures and images for: Prognostic utility of preoperative inflammatory markers in patients with intrahepatic cholangiocarcinoma after hepatic resection: A systematic review and meta‐analysis
Source: Cancer Med. 2022 Jun 12;12(1):99–110. doi: 10.1002/cam4.4935 (PMC9844628; doi:10.1002/cam4.4935)

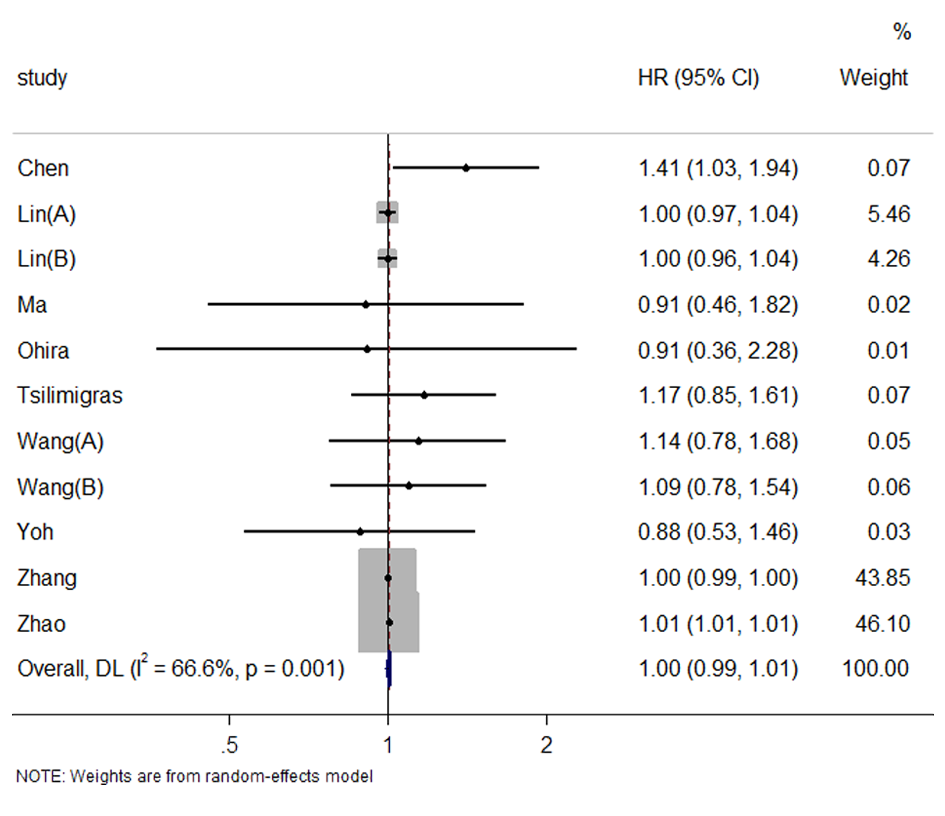

Supplement: Supplementary file 1 — Figure S1 [file CAM4-12-99-s004.tif]

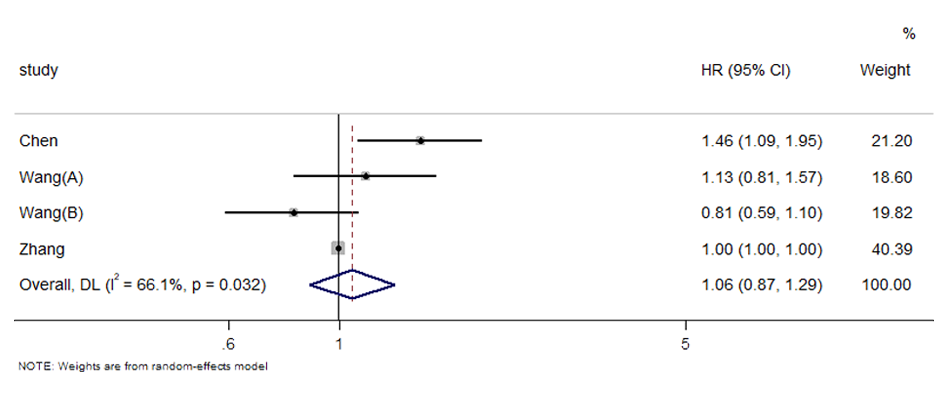

Supplement: Supplementary file 2 — Figure S2 [file CAM4-12-99-s001.tif]

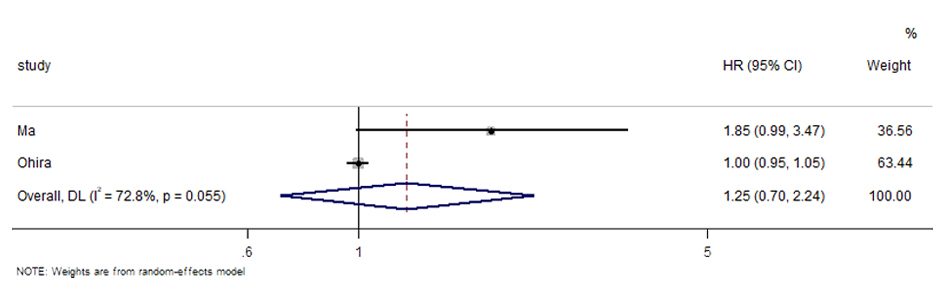

Supplement: Supplementary file 3 — Figure S3 [file CAM4-12-99-s002.tif]

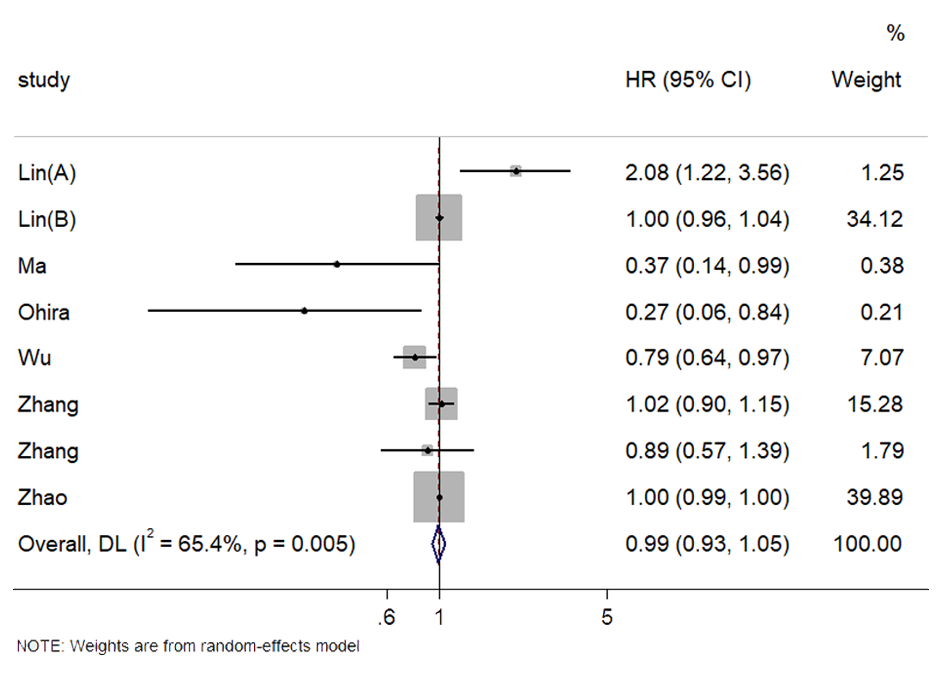

Supplement: Supplementary file 4 — Figure S4 [file CAM4-12-99-s005.tif]

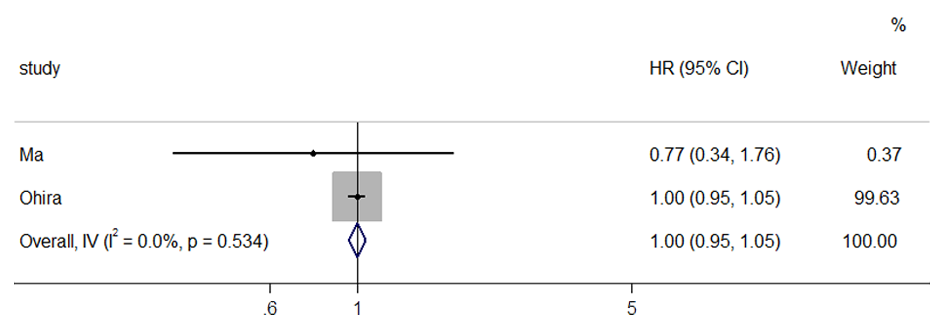

Supplement: Supplementary file 5 — Figure S5 [file CAM4-12-99-s003.tif]
