# Supplementary material for: Prognostic utility of preoperative inflammatory markers in patients with intrahepatic cholangiocarcinoma after hepatic resection: A systematic review and meta‐analysis
Source: Cancer Med. 2022 Jun 12;12(1):99–110. doi: 10.1002/cam4.4935 (PMC9844628; doi:10.1002/cam4.4935)
Supplement: Supplementary file 6 — File S1 [file CAM4-12-99-s006.docx]

Search strategy for PubMed

#1 ((((("Cholangiocarcinoma"[Mesh]) OR (((((((((((((Cholangiocarcinomas[Title/Abstract]) OR (Cholangiocellular Carcinoma[Title/Abstract])) OR (Carcinoma, Cholangiocellular[Title/Abstract])) OR (Carcinoma, Cholangiocellular[Title/Abstract])) OR (Cholangiocellular Carcinomas[Title/Abstract])) OR (Extrahepatic Cholangiocarcinoma[Title/Abstract])) OR (Cholangiocarcinoma, Extrahepatic[Title/Abstract])) OR (Cholangiocarcinomas, Extrahepatic[Title/Abstract])) OR (Extrahepatic Cholangiocarcinomas[Title/Abstract])) OR (Intrahepatic Cholangiocarcinoma[Title/Abstract])) OR (Cholangiocarcinoma, Intrahepatic[Title/Abstract])) OR (Cholangiocarcinomas, Intrahepatic[Title/Abstract])) OR (Intrahepatic Cholangiocarcinomas[Title/Abstract]))) OR (("Biliary Tract Neoplasms"[Mesh]) OR (((((((((Biliary Tract Neoplasm[Title/Abstract]) OR (Neoplasm, Biliary Tract[Title/Abstract])) OR (Neoplasms, Biliary Tract[Title/Abstract])) OR (Biliary Tract Cancer[Title/Abstract])) OR (Biliary Tract Cancers[Title/Abstract])) OR (Cancer, Biliary Tract[Title/Abstract])) OR (Cancers, Biliary Tract[Title/Abstract])) OR (Cancer of the Biliary Tract[Title/Abstract])) OR (Cancer of Biliary Tract[Title/Abstract])))) OR (("Gallbladder Neoplasms"[Mesh]) OR (((((((((((((((Gallbladder Neoplasm[Title/Abstract]) OR (Neoplasm, Gallbladder[Title/Abstract])) OR (Neoplasms, Gallbladder[Title/Abstract])) OR (Cancer of Gallbladder[Title/Abstract])) OR (Gallbladder Cancers[Title/Abstract])) OR (Gallbladder Cancer[Title/Abstract])) OR (Cancer, Gallbladder[Title/Abstract])) OR (Cancers, Gallbladder[Title/Abstract])) OR (Gall Bladder Cancer[Title/Abstract])) OR (Bladder Cancer, Gall[Title/Abstract])) OR (Bladder Cancers, Gall[Title/Abstract])) OR (Cancer, Gall Bladder[Title/Abstract])) OR (Cancers, Gall Bladder[Title/Abstract])) OR (Gall Bladder Cancers[Title/Abstract])) OR (Cancer of the Gallbladder[Title/Abstract])))) OR (("Bile Duct Neoplasms"[Mesh]) OR (((((((((Bile Duct Neoplasm[Title/Abstract]) OR (Neoplasm, Bile Duct[Title/Abstract])) OR (Neoplasms, Bile Duct[Title/Abstract])) OR (Bile Duct Cancer[Title/Abstract])) OR (Bile Duct Cancers[Title/Abstract])) OR (Cancer, Bile Duct[Title/Abstract])) OR (Cancers, Bile Duct[Title/Abstract])) OR (Cancer of the Bile Duct[Title/Abstract])) OR (Cancer of Bile Duct[Title/Abstract]))))

 #2 (((((((("Blood Platelets"[Mesh]) OR (((((((Blood Platelet[Title/Abstract]) OR (Platelet, Blood[Title/Abstract]))OR(Platelets, Blood[Title/Abstract])) OR (Thrombocytes[Title/Abstract])) OR(Thrombocyte[Title/Abstract])) OR (Platelets[Title/Abstract])) OR (Platelets[Title/Abstract]))) OR (("Monocytes"[Mesh]) OR (Monocyte[Title/Abstract]))) OR ((((((("Inflammation"[Mesh]) OR ((((Inflammations[Title/Abstract]) OR (Innate Inflammatory Response[Title/Abstract])) OR (Inflammatory Response, Innate[Title/Abstract])) OR (Innate Inflammatory Responses[Title/Abstract]))) OR (("Neutrophils"[Mesh]) OR (((((((((((((Neutrophil[Title/Abstract]) OR (Leukocytes, Polymorphonuclear[Title/Abstract])) OR (Leukocyte, Polymorphonuclear[Title/Abstract])) OR (Polymorphonuclear Leukocyte[Title/Abstract])) OR (Polymorphonuclear Leukocytes[Title/Abstract])) OR (LE Cells[Title/Abstract])) OR (Cell, LE[Title/Abstract])) OR (Cells, LE[Title/Abstract])) OR (LE Cell[Title/Abstract])) OR (Neutrophil Band Cells[Title/Abstract])) OR (Band Cell, Neutrophil[Title/Abstract])) OR (Band Cells, Neutrophil[Title/Abstract])) OR (Neutrophil Band Cell[Title/Abstract])))) OR (("Lymphocytes"[Mesh]) OR (((((Lymphocyte[Title/Abstract]) OR (Lymphoid Cells[Title/Abstract])) OR (Cell, Lymphoid[Title/Abstract])) OR (Cells, Lymphoid[Title/Abstract])) OR (Lymphoid Cell[Title/Abstract])))) OR (NLR)) OR (Neutrophil to lymphocyte ratio)) OR (neutrophil-to-lymphocyte ratio))) OR (PLR[Title/Abstract])) OR (MLR[Title/Abstract]) ) OR (Platelet to lymphocyte ratio[Title/Abstract])) OR (Lymphocyte to monocyte ratio[Title/Abstract]))

#3 (((((((((((Prognosis"[Mesh]) OR (Prognostic Factors[Title/Abstract])) OR (Factor, Prognostic[Title/Abstract])) OR (Factors, Prognostic[Title/Abstract])) OR (Prognostic Factor[Title/Abstract])) OR (overall survival[Title/Abstract])) OR (disease-free survival[Title/Abstract])) OR (progression-free survival[Title/Abstract])) OR (OS[Title/Abstract])) OR (DFS[Title/Abstract])) OR (PFS[Title/Abstract])) OR (Survival[Title/Abstract])

#1 and #2 and #3
